# Supplementary figures and images for: Comparative analysis of Spodoptera frugiperda (J. E. Smith) (Lepidoptera, Noctuidae) corn and rice strains microbiota revealed minor changes across life cycle and strain endosymbiont association
Source: PeerJ. 2024 Apr 12;12:e17087. doi: 10.7717/peerj.17087 (PMC11017975; doi:10.7717/peerj.17087)

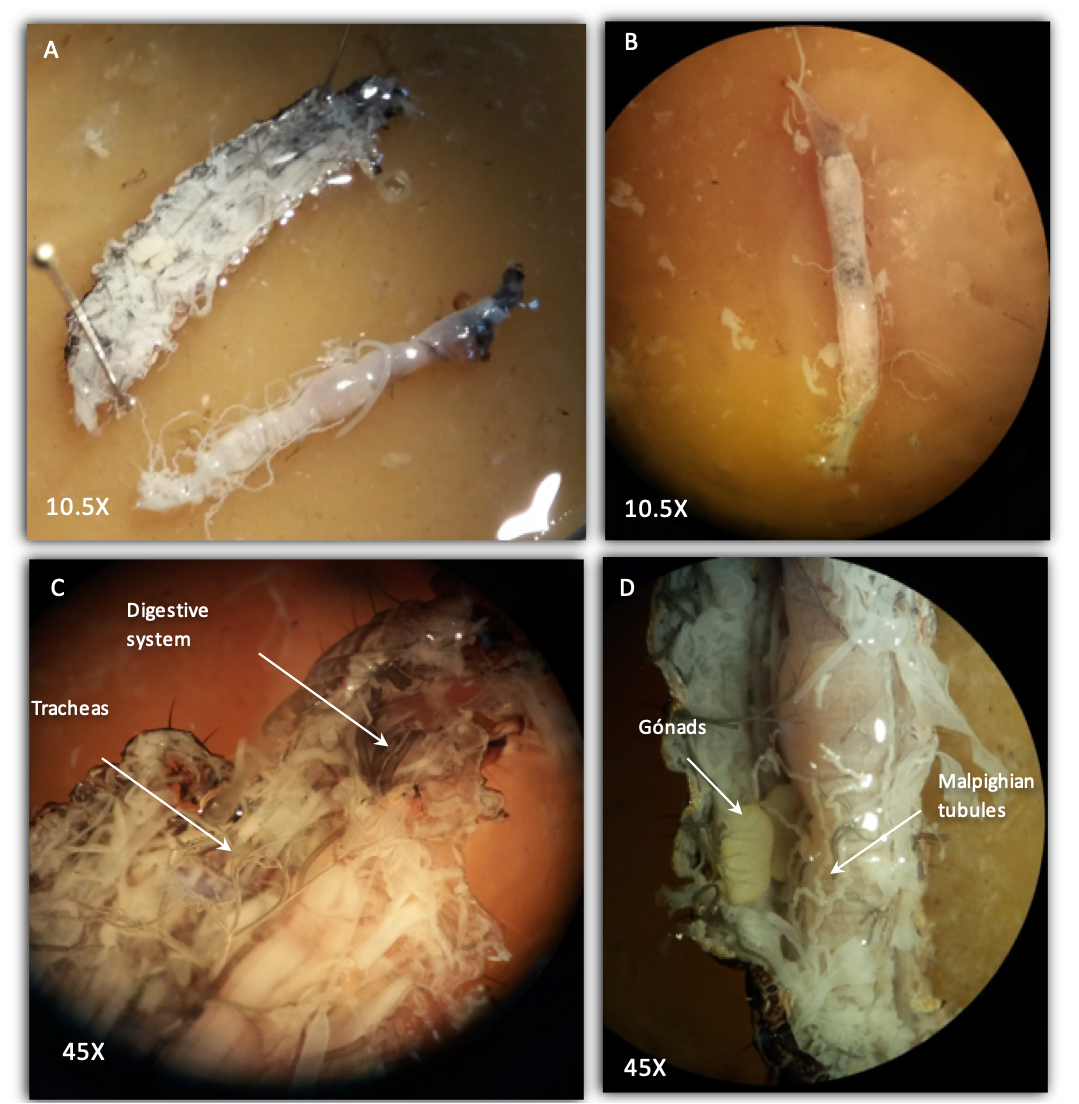

Supplement: Supplemental Information 1 — Dissection of the larvae digestive system (A and B). View of the insect’s respiratory system (C). Branches of the Malpighian tubules and the gonads are seen on the left side (D) [file peerj-12-17087-s001.png]

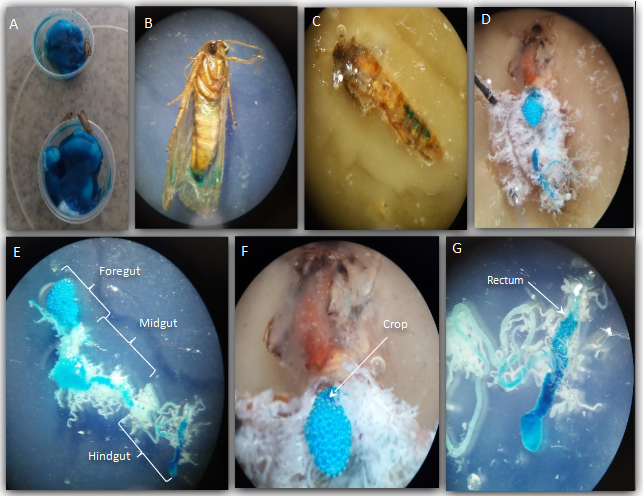

Supplement: Supplemental Information 2 — System used for feeding moths (A). The appearance of adults fed with water-honey stained with Blue No.1, ventral view (B) and external dorsal view of the insect (C). Abdomen with exposed digestive system marked with dye (D). Details of digestive regions and structures (E–G) [file peerj-12-17087-s002.png]

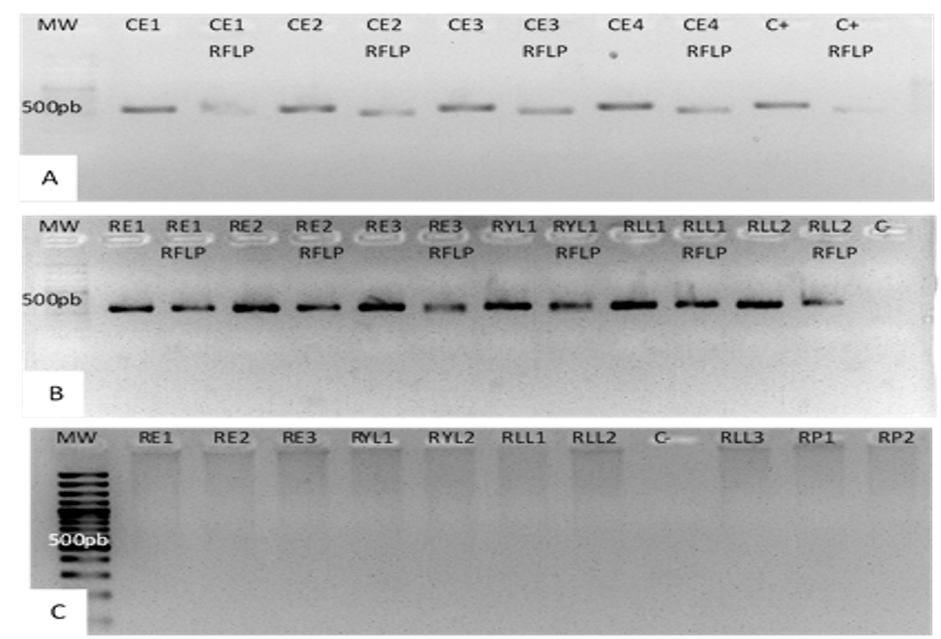

Supplement: Supplemental Information 3 — Amplification of the COI gene and its treatment with the restriction enzyme MSPI of insects collected from corn plants. All the samples were digested with the MSPI enzyme (A). Amplification of the COI gene and its treatment with the restriction enzyme MSPI of insects collected from rice plants. There was no digestion with MSPI enzyme in any of the samples (B) Amplification of the nuclear region FR of insects from rice plants. Amplification products higher than 500 bp are observed (C). [file peerj-12-17087-s003.png]

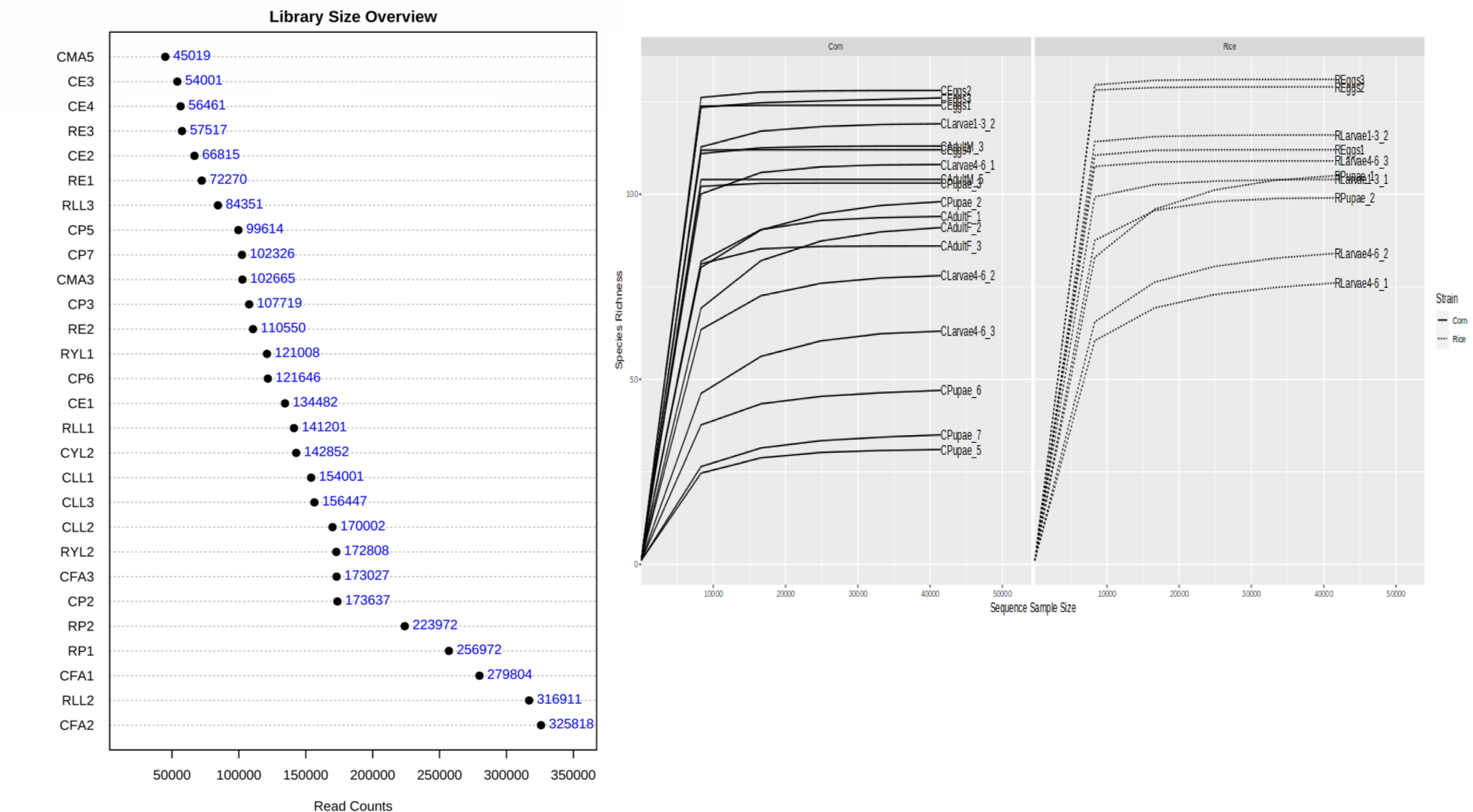

Supplement: Supplemental Information 4 [file peerj-12-17087-s004.png]

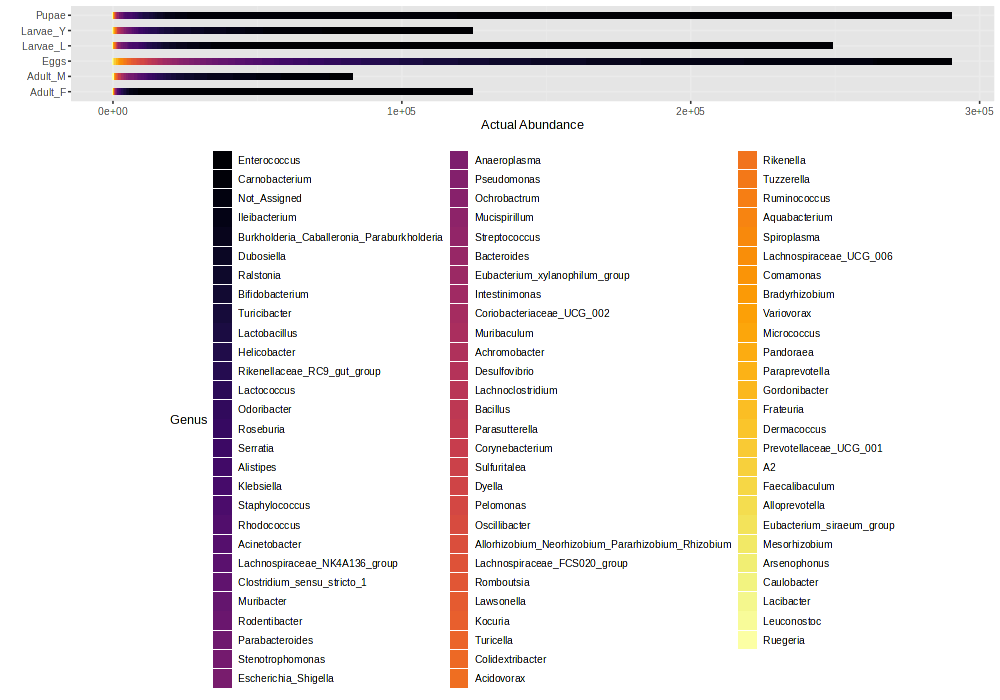

Supplement: Supplemental Information 5 [file peerj-12-17087-s005.png]

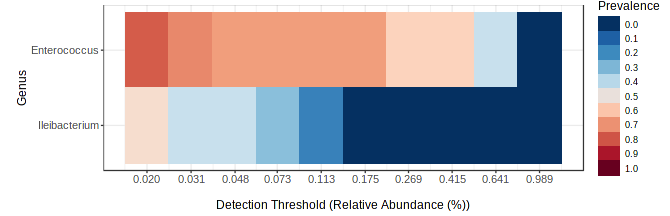

Supplement: Supplemental Information 6 [file peerj-12-17087-s006.png]

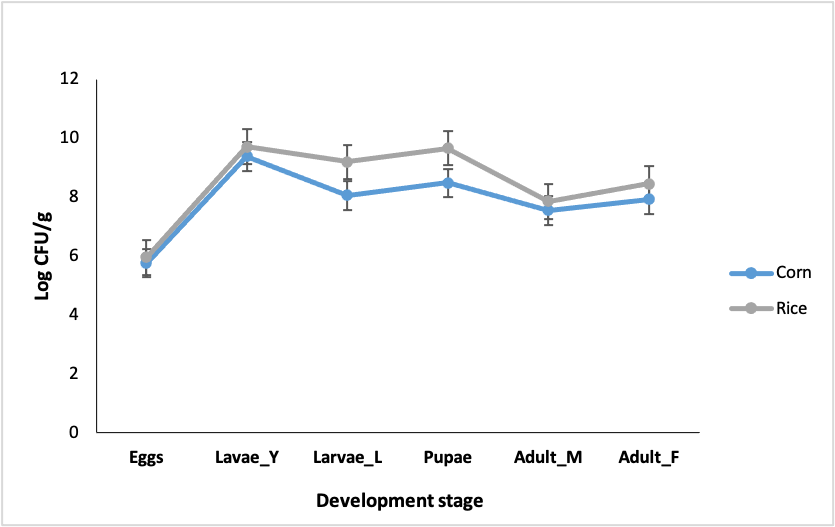

Supplement: Supplemental Information 7 [file peerj-12-17087-s007.png]

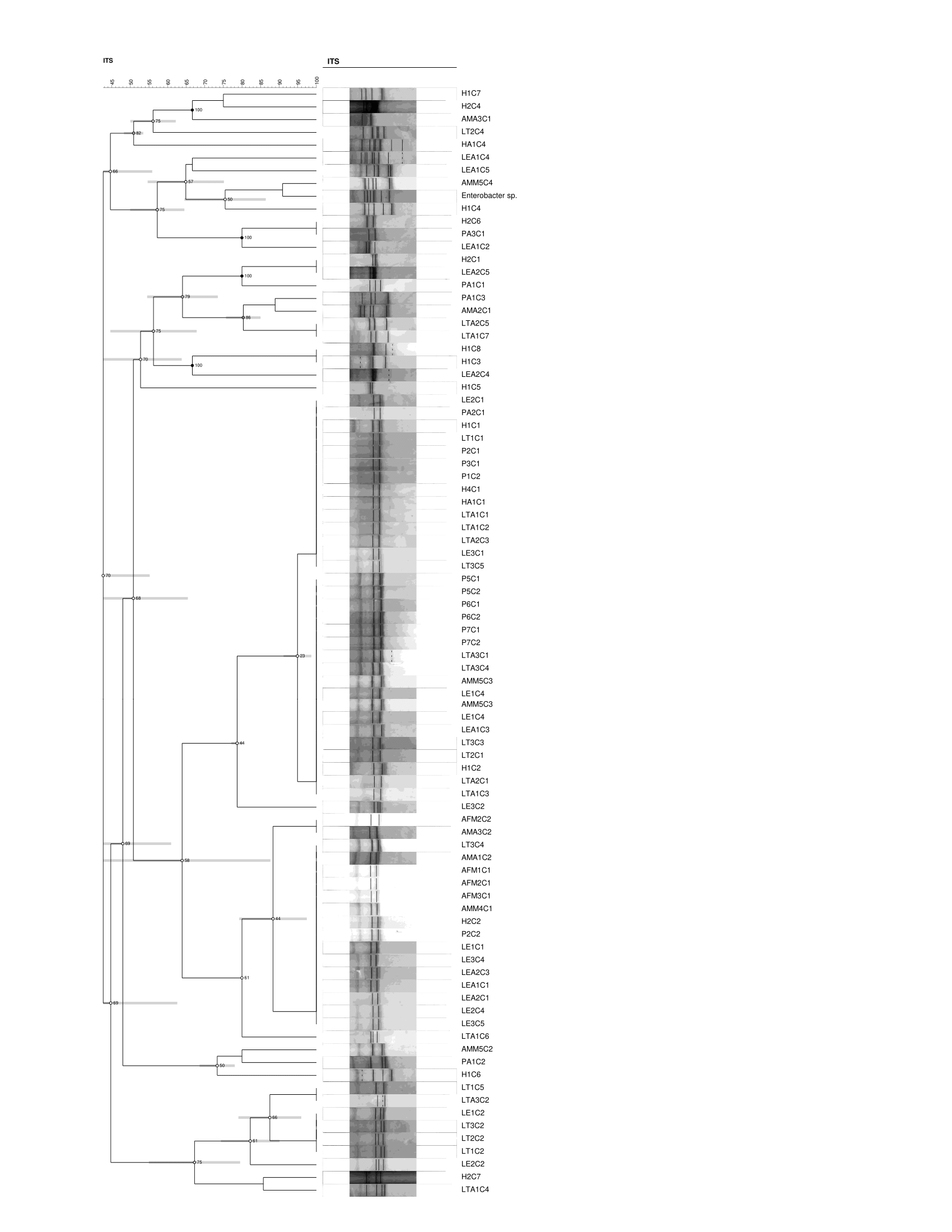

Supplement: Supplemental Information 8 [file peerj-12-17087-s008.png]
