# Supplementary material for: Comparative analysis of Spodoptera frugiperda (J. E. Smith) (Lepidoptera, Noctuidae) corn and rice strains microbiota revealed minor changes across life cycle and strain endosymbiont association
Source: PeerJ. 2024 Apr 12;12:e17087. doi: 10.7717/peerj.17087 (PMC11017975; doi:10.7717/peerj.17087)
Supplement: Supplemental Information 9 [file peerj-12-17087-s009.docx]

**Supplementary Table 1**. NGS sequencing quality data by Illumina MiSeq

|  | Input | Filtered | DenoisedF | DenoisedR | Merged | Nonchim |
| --- | --- | --- | --- | --- | --- | --- |
| Adult_F_C1 | 431064 | 316990 | 314967 | 315046 | 300308 | 289311 |
| Adult_F_C2 | 473685 | 358479 | 355243 | 356698 | 339650 | 329316 |
| Adult_F_C3 | 282111 | 200323 | 198550 | 199380 | 189720 | 180769 |
| Adult_M_C3 | 205538 | 150705 | 148349 | 148912 | 140809 | 126765 |
| Adult_M_C5 | 525868 | 359015 | 356802 | 356290 | 336963 | 67399 |
| Eggs_C1 | 364105 | 256060 | 254464 | 253671 | 238447 | 220505 |
| Eggs_C2 | 171829 | 124632 | 122614 | 122292 | 110946 | 100611 |
| Eggs_C3 | 129742 | 91850 | 90326 | 90095 | 82571 | 75332 |
| Eggs_C4 | 166248 | 116208 | 114882 | 114799 | 91235 | 84822 |
| Eggs_R1 | 129540 | 102876 | 102019 | 101496 | 92204 | 85237 |
| Eggs_R2 | 352095 | 213712 | 212137 | 211091 | 188340 | 175724 |
| Eggs_R3 | 134021 | 93237 | 91863 | 91507 | 85135 | 78480 |
| Larvae_Y_C2 | 314767 | 196183 | 194627 | 194675 | 184363 | 155162 |
| Larvae_Y_R1 | 255453 | 169753 | 168346 | 168219 | 158125 | 129625 |
| Laevae_Y_R2 | 471525 | 305108 | 303153 | 302465 | 283812 | 257944 |
| Larvae_L_C1 | 290845 | 194973 | 193690 | 193610 | 184681 | 160819 |
| Larvae_L_C2 | 243939 | 187014 | 186187 | 186199 | 180481 | 170688 |
| Larvae_L_C3 | 226755 | 170694 | 170229 | 170115 | 166682 | 156766 |
| Larvae_L_R1 | 186194 | 149995 | 149519 | 149520 | 146093 | 142203 |
| Larvae_L_R2 | 475376 | 357699 | 356934 | 356171 | 346996 | 318680 |
| Larvae_L_R3 | 186291 | 115838 | 114497 | 114447 | 107508 | 98249 |
| Pupae_C2 | 244509 | 186492 | 185620 | 185921 | 180087 | 176232 |
| Puepae_C3 | 215654 | 149494 | 148340 | 148342 | 140752 | 126444 |
| Pupae_C5 | 129224 | 103432 | 103100 | 103029 | 101327 | 99735 |
| Pupae_C6 | 165973 | 128113 | 127601 | 127325 | 124601 | 121928 |
| Pupae_C7 | 143094 | 113605 | 113244 | 113283 | 111378 | 102437 |
| Pupae_R1 | 389104 | 288784 | 288039 | 287835 | 279472 | 262041 |
| Pupae_R2 | 369546 | 265321 | 264289 | 264032 | 252947 | 232934 |
